# A web-based library consult service for evidence-based medicine: Technical development

**DOI:** 10.1186/1472-6947-6-16

**Published:** 2006-03-16

**Authors:** Alan Schwartz, Gregory Millam, the UIC LCS Investigators

**Affiliations:** 1 Department of Medical Education, University of Illinois at Chicago, Chicago, USA; 2 Department of Pediatrics, University of Illinois at Chicago, Chicago, USA

## Abstract

**Background:**

Incorporating evidence based medicine (EBM) into clinical practice requires clinicians to learn to efficiently gain access to clinical evidence and effectively appraise its validity. Even using current electronic systems, selecting literature-based data to solve a single patient-related problem can require more time than practicing physicians or residents can spare. Clinical librarians, as informationists, are uniquely suited to assist physicians in this endeavor.

**Results:**

To improve support for evidence-based practice, we have developed a web-based EBM library consult service application (LCS). Librarians use the LCS system to provide full text evidence-based literature with critical appraisal in response to a clinical question asked by a remote physician. LCS uses an entirely Free/Open Source Software platform and will be released under a Free Software license. In the first year of the LCS project, the software was successfully developed and a reference implementation put into active use. Two years of evaluation of the clinical, educational, and attitudinal impact on physician-users and librarian staff are underway, and expected to lead to refinement and wide dissemination of the system.

**Conclusion:**

A web-based EBM library consult model may provide a useful way for informationists to assist clinicians, and is feasible to implement.

## Background

Despite the growing availability of evidence-based medicine (EBM) training programs for practicing physicians and increasing emphasis on EBM in medical student and resident education, research indicates that even those clinicians who are most enthusiastic about EBM generally rely more on traditional information sources, such as consultation with respected colleagues, than on EBM-related sources [1]. In reviewing the teaching of EBM as an educational endeavor, several reviews have concluded that evidence-based practice may be difficult for physicians [2-6].

EBM requires that clinicians learn new skills, including how to formulate questions about their patients that can be answered in the medical literature, how to search the clinical research literature for potentially relevant research reports, how to critically appraise the research design and analysis methods in order to determine the validity of reported results and their applicability to their patients, and how to use valid results appropriately in making clinical decisions. Such skills, the foundations of which lie in biostatistics, epidemiology, and library and information science, typically are not part of the armamentarium of medical school faculty and hence, are not easily promoted in medical students and residents. Even for those physicians who have acquired some skills and are committed to using them, access to the clinical evidence is far from easy, with searches often yielding either irrelevant citations or none at all [7]. While using current electronic systems, physicians can discover that finding and selecting literature-based data to solve a single patient-related problem can easily require an hour or more [8], time that neither practicing physicians nor busy residents typically have. Further, clinicians often cite discomfort with assessing the methodological quality of clinical studies as a major deterrent to use of EBM [1].

Since the creation, some 30 years ago, of the clinical medical librarianship program (CML) by Gertrude Lamb, several studies have demonstrated the effectiveness of an active partnership between clinicians and clinical librarians [9,10]. Clinical librarianship programs add to clinicians' knowledge most of the time, affect clinical decisions a substantial proportion of the time, and even improve certain outcomes, such as length of stay [11]. Kuller, et al. [12] found that librarians recognize and select relevant articles as effectively as physicians. Despite general acceptance of the desirability of CML programs, some studies indicate physician concerns, including possible misunderstanding by librarians of clinical questions, inadequate knowledge of medical terminology, and skepticism about librarians' ability to judge the quality of clinical research [13-15].

Increasingly, library authorities emphasize the importance of rethinking medical librarians' roles in the providing of medical information. Klein and Ross [16] call for value-added service roles, such as quality filtering; Guise [17] argues that "...librarians should read the full text of the most pertinent articles retrieved by their searches, identify and extract the information relevant to the clinical question at hand, and write a brief essay...describing their findings." To ensure that they can do this, librarians should "seek instruction in the techniques of clinical trials", "study...evidence-based medicine", and receive "mentored instruction and practice in searching, retrieving, filtering and summarizing information." Davidoff and Florance [11] echo Guise and propose a new role, the "informationist", in which clinical librarians, in addition to performing their traditional search role, should be taught to evaluate and synthesize medical information in a timely and effective manner. Plutchak [18] revisits and reinforces this argument in an editorial that accompanies the report of the 2002 Informationist Conference [19]. Byrd [20] offers an analogy with changes in the profession of pharmacy and the role of pharmacists as clinical team members.

In an effort to actualize these recommendations, improve support for evidence-based practice, and increase physicians' use of EBM, a team of physicians, medical educators, programmers and librarians developed a web-based EBM library consult service application (LCS). The LCS system is designed to provide full text evidence-based literature with critical appraisal in response to a clinical question asked by a physician who may be at a remote or rural site. The first year of the LCS project focused on software development and reference implementation. During the upcoming two years, the service will be provided to two clinical departments at the University of Illinois at Chicago (UIC), and evaluated by its users using multiple methods. In addition, a second, separate LCS system will be implemented and evaluated at the University of Illinois at Chicago Peoria campus targeting rural community physicians.

The idea of providing clinicians with a consult service focused on addressing clinical decisions with evidence has been practiced in other settings. Two particularly notable examples include the Clinical Decision Consultation Service at the New England Medical Center, which provides decision analysis consultations by physician-analysts to clinicians with a turnaround as fast as 24 hours for urgent cases [21], and the Clinical Informatics Consult Service (CICS) of the Eskind Biomedical Library at Vanderbilt University Medical Center, which integrates librarians into medical rounds where they can select and appraise evidence [15,17,22] The LCS approach is similar to the CICS, but uses the world-wide web to extend the reach of the service.

## Implementation

### Implementation sites

The reference implementation of the LCS is at the Library of Health Sciences (LHS) at the UIC College of Medicine. The UIC College of Medicine is the largest medical school in the United States, and has a faculty of four thousand (full and part time, and volunteers) at four locations across the state: Chicago, Peoria, Rockford, and Urbana-Champaign. Nearly 1300 medical students are educated each year.

The primary clinical site for UIC is the University of Illinois Medical Center at Chicago, a large urban medical center that serves a socioeconomically diverse population. Approximately 300,000 outpatient visits are made each year. Patients are predominantly African-American (51%), Hispanic (24%), and White (21%), and represent a fairly uniform distribution of ages from newborn to over-65. Most patients are enrolled in HMOs or Medicaid.

The University of Illinois Medical Center is connected to a high-speed university FDDI network with OC3 Internet connectivity. The primary information resources for clinicians at UIC are provided by LHS. LHS supports the College of Medicine's mission of teaching, research and service at its main campus in Chicago as well as at the three sites of the medical school located in Peoria, Rockford, and Urbana where site libraries are also staffed by health sciences librarians. The University Library currently provides access to over 20,000 electronic journals, 16,000 current serial titles and 1.9 million volumes. LHS serves as the Regional Medical Library for the ten-state Greater Midwest Region under a contract awarded by the U.S. National Library of Medicine.

The reference implementation of LCS serves the residents and faculty of the Departments of Pediatrics and Family Medicine at the UIC College of Medicine. There are approximately 78 residents and 64 full-time physician faculty in these departments. The reference implementation is staffed by six clinical librarians and library residents at UIC.

### Information flow

**Figure 1:**
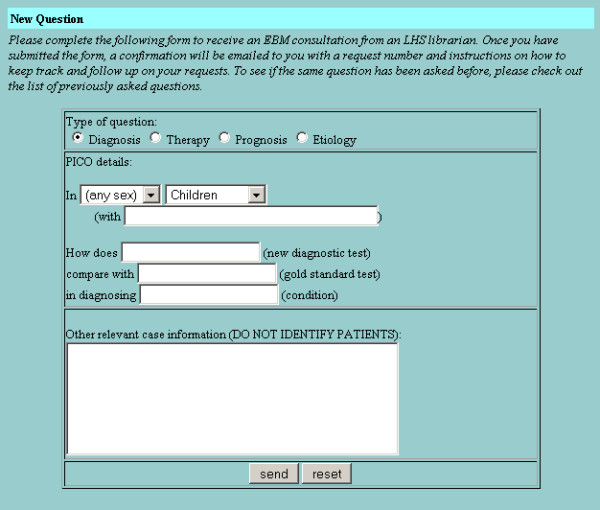
Dynamic web form for submitting an answerable clinical question about diagnosis. Changing question type with the radio button dynamically changes the prompts in the form to elicit the key elements of other types of clinical questions.

**Figure 2:**
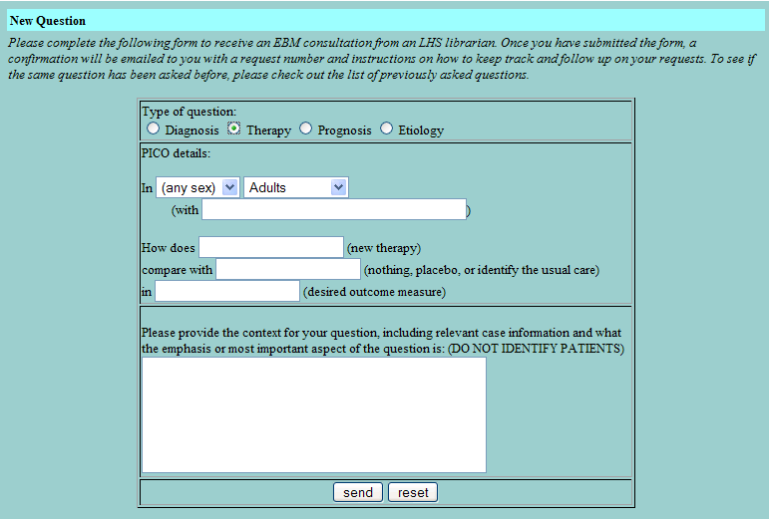
Dynamic web form for submitting an answerable clinical question about therapy.

**Figure 3:**
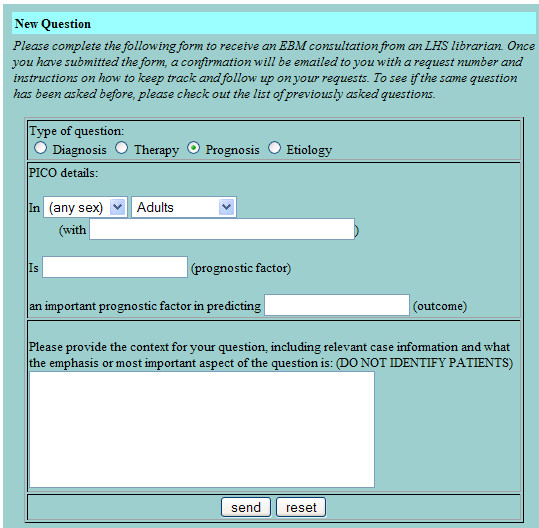
Dynamic web form for submitting an answerable clinical question about prognosis.

**Figure 4:**
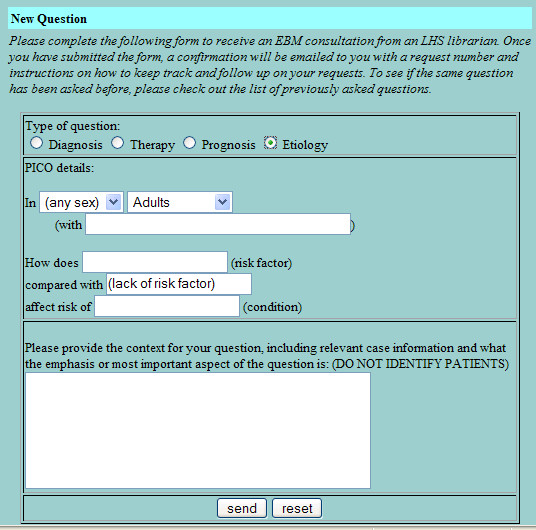
Dynamic web form for submitting an answerable clinical question about etiology.

The basic design of the LCS is intended to mirror and enhance the way that clinicians and clinical librarians naturally interact. The clinician submits a query to the LCS using a dynamic web form that prompts for the elements of an answerable clinical question [23] (Figures 1, 2, 3, 4). LCS compares the question keywords to those of other questions that have been answered recently and, if any such questions are found, asks the clinician whether these answers are acceptable or whether the question should be submitted for a new answer. If submitted, LCS stores the question in its database, emails the clinician to notify him/her that the question has been received, and emails one or more designated librarians to notify them that a new question has been submitted. At this stage, LCS may also apply "triage" rules to questions, e.g. notifying clinicians when the number of unanswered questions per participating librarian is particularly heavy and response time may be slower.

The notified librarians are responsible for assigning the question to a librarian. Because any number of librarians can be notified when questions are received, LCS supports several different models of library staffing. For example, a single librarian can be designated to be notified of new questions, and given the responsibility of assigning each question to a suitable librarian, or all librarians can be notified of new questions, and any librarian can choose to answer. Once a librarian is assigned to a question, the librarian (and, optionally, the clinician) is notified by email.

The librarian may need clarification of the question by the clinician, which can take place either through LCS or without the mediation of LCS. The assigned librarian may submit a request for clarification through LCS, which is stored with the question and emailed to the clinician, or may contact the clinician directly by phone.

**Figure 5:**
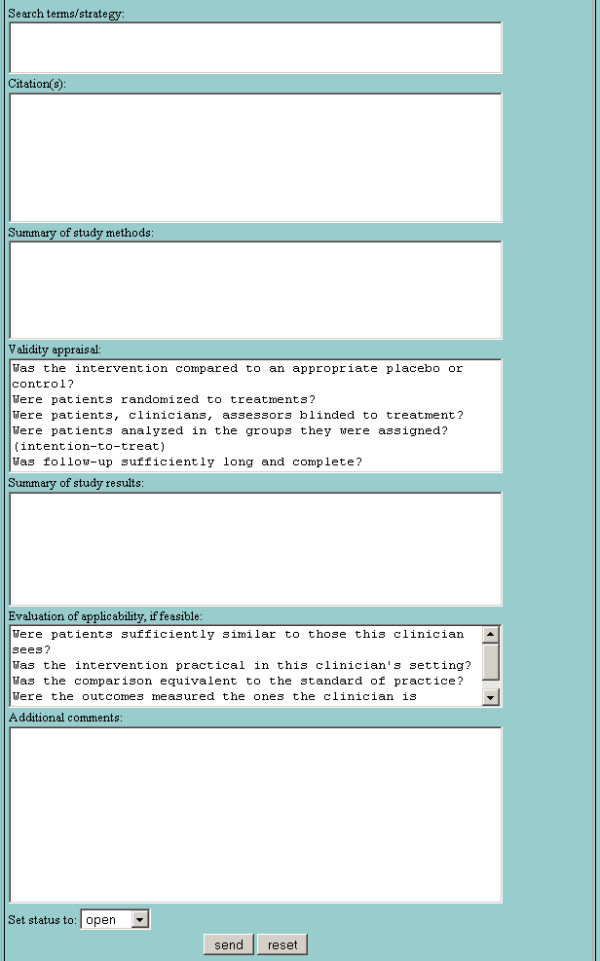
The LCS response form for librarians to submit answers to questions. The form mimics a "critically-appraised topic" form, with response queues pre-inserted in some fields.

The assigned librarian then answers the question by performing a search using library resources (including, perhaps, the LCS database of previous questions), and fills out a response form that mimics the "Critically Appraised Topic" (CAT) format recommended by Sauve, et al. [24] (Figure 5)

**Figure 6:**
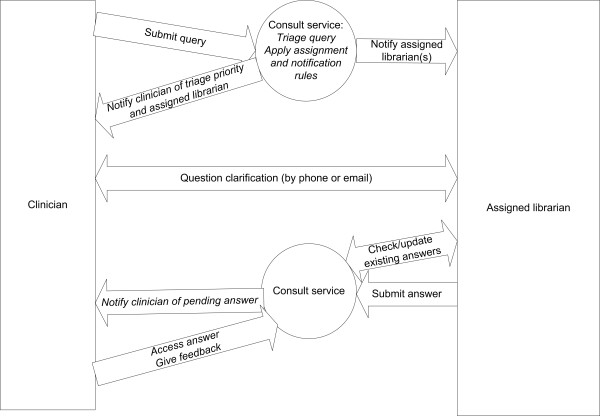
Process flow diagram for the Library Consult Service, illustrating interactions between clinician, consult service, and librarian.

**Figure 7:**
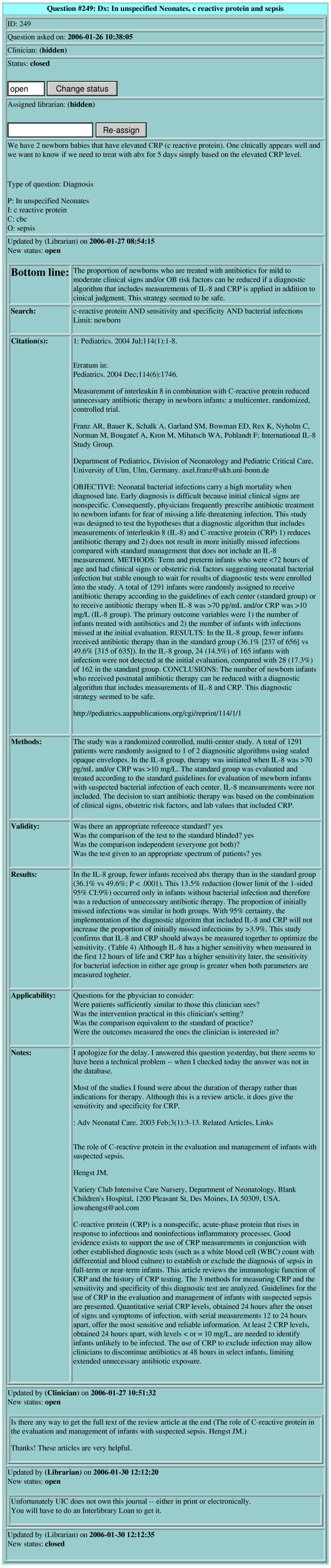
A completed LCS question with responses.

LCS emails the completed response to the clinician, who can also retrieve it by logging into LCS. Clinicians are reminded (in the email and on login) to provide feedback and rate the quality of the answers they receive; once responses are rated, the interaction (question, response, feedback) is considered complete. LCS system procedures are illustrated in Figure 6. The LCS question page for a question that has completed the entire cycle is show in Figure 7.

### Platform

For both practical and philosophical reasons, the system was designed to be licensed as free/open source software (FOSS) [25,26] and to be built using FOSS components. LCS is written in the Ruby programming language, and was developed to run under the Linux operating system, Apache web server, and MySQL database.

Linux was chosen because it runs on a variety of hardware platforms, providing a consistent environment. The reference hardware platforms include a Sun Ultra 5 workstation running Debian GNU/Linux 3.1 for SPARC and a Dell workstation running Debian GNU/Linux 3.1 for Intel x86 hardware [27].

The Apache web server similarly is available for a wide range of operating systems. Although the LCS implementation uses Apache 1.3.33[28] and the mod_ruby module[29] to improve the speed of processing, there is nothing in the LCS software that makes any special requirements of the web server other than the ability to execute Common Gateway Interface (CGI) scripts.

MySQL 4.1[30] was chosen as the database server for the reference implementations because it provides a fast, stable, and portable relational database that implements a large subset of ANSI SQL 99.

Ruby[31] is a completely object-oriented scripting language with strong exception handling features. Ruby was chosen because objects provide a convenient representation for most of the components of the LCS system, such as users, questions, and interface components. LCS requires Ruby 1.8 or later.

### Database design

**Figure 8:**
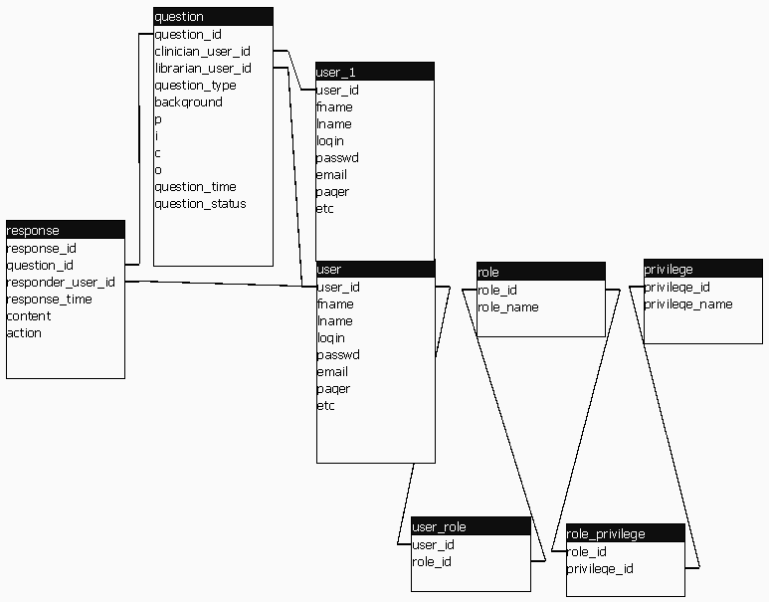
Relational structure of primary entities in the Library Consult Service database. The "user" and "user_1" boxes represent two instances of the same table of users.

The LCS database uses a relatively simple relational structure. The primary database entities include users, questions, and responses (Figure 8); secondary entities, user roles and privileges, user customization options, etc. are more numerous. A user is a librarian or a clinician; a question is an answerable clinical question, submitted by a user; a response is text associated with a question and submitted by a user (e.g., a librarian's answer to a question, a physician's addendum to a question or evaluation of the usefulness of the librarian's answer). A question, together with its chain of responses in chronological order, constitutes a complete system interaction.

### User interface

The key interface design features for LCS include a familiar visual design that emphasizes usability, personalization of web pages, and online context-sensitive help. The primary interface consists of a navigation menu on the left side of the screen and one or more "blocks" of content on the right side of the screen that change as users select options from the navigation menu, a design similar to that employed by PUBMED^® ^[32] By default, a single block of content is presented on the right side of the screen for clinicians, and two blocks of content (i.e., the two most recently requested options) are presented for librarians, who must often refer to multiple types of content at once. Individual users can increase or decrease the number of blocks they wish to see at once.

Personalization is a critical element of the design, and serves to make the information load manageable for system users by clearly distinguishing between information targeted to the user and other information (targeted to other users, or more general in nature). On login, the service provides each user (librarian or physician) with a personal block, customized based on past interactions with the system. For example, a librarian sees a queue of queries assigned and unassigned. Similarly, physician users of the system receive a personalized list of their own pending queries. Users can also access responses to queries of others, with the questioner's identity masked.

Online context-sensitive help is built into the system, and can be extended by the system administrator without knowledge of Ruby. Each page presented to users may have its own help, which is implemented using a Wiki-like collection of keywords associated with HTML pages.

### System programming

As discussed above, LCS is programmed primarily in the Ruby programming language, with database queries issued using Structured Query Language (SQL). Some web pages also use Javascript for browser detection or dynamic form generation. The Ruby source code is organized around several key objects that encapsulate the functionality of the database (User, Question) and the interface (Page, Block, Action), and associated methods that implement application logic (e.g., role-checking and session management are methods of User, logic for handling responses are methods of Question). The Ruby PageTemplate library is employed throughout to separate the page display from the application logic and make it simpler to customize or modify the visual design; each web page, block, or email message generated by the system is represented by a template file that can be edited without knowledge of Ruby. This is particularly important in the early stages of system implementation, as system modification is expected in response to feedback from librarian and clinician users. It also makes it simpler for future installations of the system by others to customize the look of the system to fit local needs.

Releases of the LCS software are available from the project's SourceForge project page [33].

## Discussion

During the next two years of the project, the reference implementation of the LCS will be evaluated for feasibility, usefulness, and educational impact. In addition, a second LCS will be implemented and evaluated at the UIC Library of the Health Sciences – Peoria with a cohort of twenty rural community physicians.

### Feasibility

Feasibility of the service will be evaluated by documenting the processes of development, training, implementation, and evaluation. The documentation will consider each project phase individually and include a detailed log of the activities required during the phase and the resources required (hardware, software, expertise, programmer time, librarian time, user time). This evaluation will be useful for establishing the replicability of the service at other sites and for estimating how the service might scale to support larger numbers of users.

### Usefulness

Usefulness measures include frequency and type of use, attitudes toward the service, evaluation of individual responses to questions, and a comparison of the quality of responses produced by the service to those produced by the physician-users themselves. The frequency of use of the service is measured and subtotaled on a monthly basis. Several relevant metrics are computed on a quarterly basis, such as the average daily question load per librarian, average time from question to response, and distribution of question types and sources of evidence returned.

User and staff attitudes toward the consult service are assessed every six months using a locally-developed assessment instrument. The primary approach in this evaluation is within-subject, as we anticipate both substantial individual differences and, in the case of staff, small sample sizes. Changes in the attitude toward the consult service subscale will be modeled using hierarchical linear modeling of individual change parameters[34]. This approach offers flexibility (e.g. modeling growth using nonlinear or piecewise linear functions rather than a single linear effect) as well as methods for handling missing administrations.

The value of the specific information provided by the service is rated by users at the time that they receive the information. On the web page that presents the consult results to physicians, they are asked to rate the relevance of the evidence received, the quality of the interpretation, the likelihood that the evidence will have an impact on the patient's care, and the likelihood that the evidence will affect how they treat patients in the future, using 7-point category rating scales. They are also offered the opportunity to provide open-ended feedback about the consult. These data will be examined every six months.

Finally, an evaluation study is planned to provide convergent evidence about the value of the consult service in locating information. In this study, a representative subsample of the physician users will be asked to perform their own searches in response to answerable clinical questions (submitted by other users to the consult service within the last six months) and select the article(s) they would read to answer the question. Searching time required will be recorded, and the citations returned will be compared to those returned earlier by the consult service. Differences will be characterized qualitatively by the investigators. In addition, a subsample of responses by librarians will be compared to critical appraisals of the same articles by the investigators to evaluate and assure the quality of the librarian appraisals.

### Educational impact

Educational impact of the LCS on EBM skills and attitudes of both physicians and librarians will be evaluated regularly during the upcoming years of the project. Assessment tools have been developed to measure critical appraisal skills [35], as well as ability to formulate answerable clinical questions. Changes in scores on the skill assessments will be modeled using hierarchical linear modeling of individual change parameters[34] and traditional repeated-measures multivariate analysis of variance approach, as with attitudes toward the consult service discussed above. Amount of use of the consult service (number of questions submitted by the physician) will be introduced as a covariate in the physician skill modeling.

## Conclusion

The web-based Library Consult Service represents a natural evolution of the processes of evidence-based practice. Its release as, and reliance on, Free/Open Source Software offers administrators the freedom to modify the system to suit their needs and is likely to be cost-effective for experimentation by libraries. As an information system, it connects clinicians who have patient-oriented information needs with clinical librarians who have expertise in search and appraisal of the medical literature. It may also serve as an effective platform for EBM education and research on evidence-based clinical practice.

## Availability and requirements

Project name: EBM Library Consult Service

Project home page: 

Operating system(s): Linux

Programming language: Ruby

Other requirements: Ruby 1.8 with PageTemplate 2.1.6, MySQL 4.1 or higher

License: GNU GPL version 2 or later

## Competing interests

The author(s) declare that they have no competing interests.

## Authors' contributions

AS conceived of the project, participated in its design and coordination, and drafted the manuscript. GM participated in the design of the system, performed system programming, and helped to draft the manuscript. All authors read and approved the final manuscript.

The UIC LCS Investigators consist of Alan Schwartz, Ph.D. (Departments of Medical Education and Pediatrics), Jordan Hupert, M.D. (Department of Pediatrics), Carol Scherrer, M.A.L.S. (Library of Health Sciences), Karen Connell, M.S. (Department of Family Medicine), Jerry Niederman, M.D., M.P.H. (Department of Pediatrics), and Josephine Dorsch, M.A.L.S. (Library of Health Sciences), all of whom meet the Vancouver criteria for authorship of this paper. Gregory Millam was responsible for the majority of the programming of the LCS itself and also co-authored this paper.

## Pre-publication history

The pre-publication history for this paper can be accessed here:


